# Supplementary figures and images for: Photoinduced electron transfer detection method for identifying UGT1A1*28 microsatellites
Source: PLoS One. 2023 Aug 3;18(8):e0289506. doi: 10.1371/journal.pone.0289506 (PMC10399816; doi:10.1371/journal.pone.0289506)

NO. \_\_\_\_\_

DATE \_\_\_\_\_

**A**

17mer

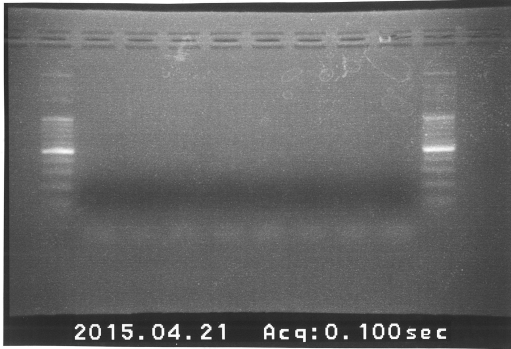

H G F E D C B A

**B**

20mer

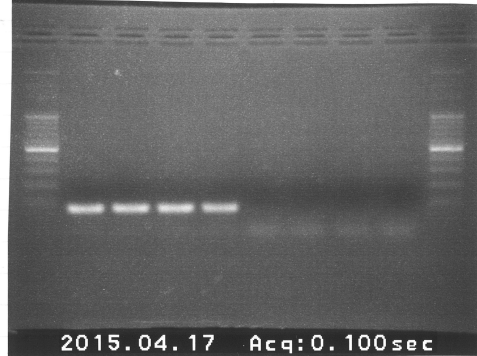

H G F E D C B A

**C**

21mer

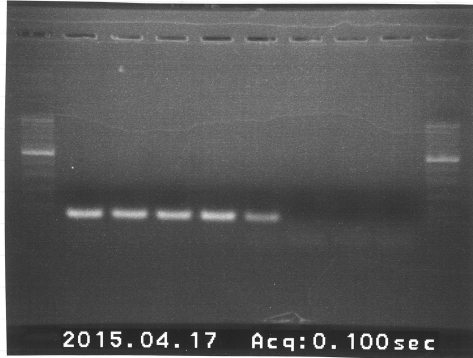

H G F E D C B A

26mer

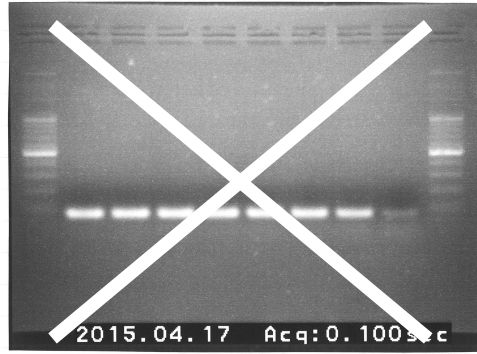

H G F E D C B A

37mer

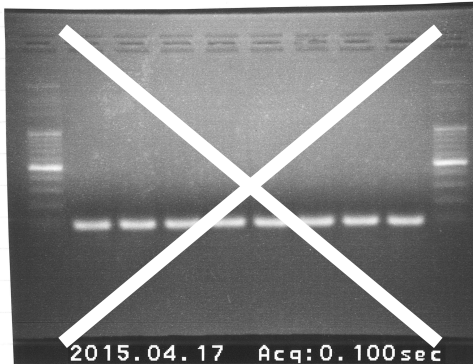

H G F E D C B A

38mer

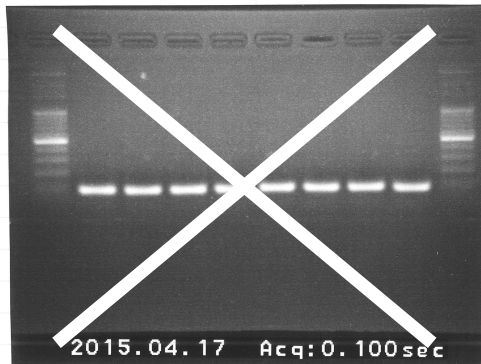

H G F E D C B A

**D**

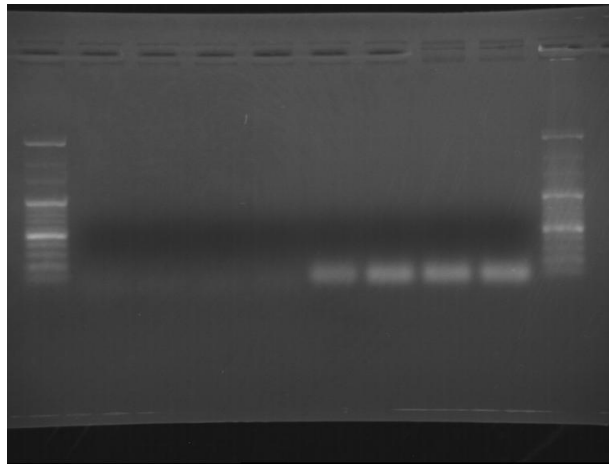

**E**

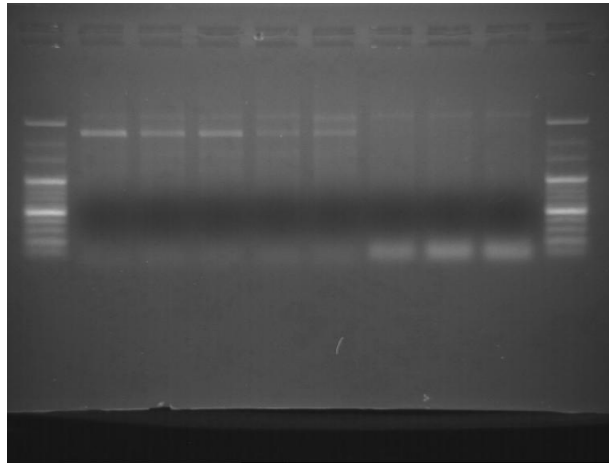

**F**

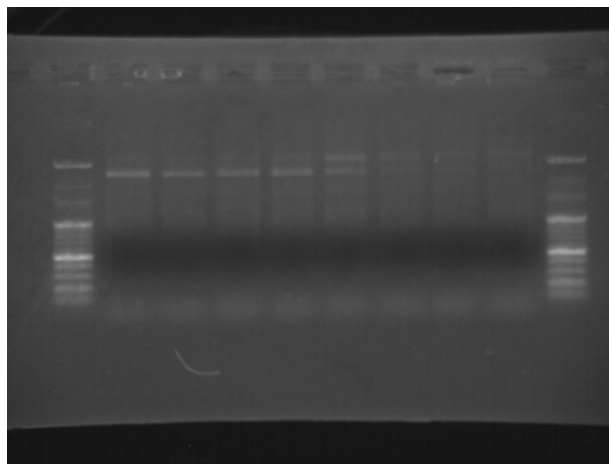

Supplement: S1 Raw images — (A) Agarose gel electrophoresis images of PCR mixtures at various annealing temperatures (30–55°C) using the forward primer 17 mer and reverse primer in Fig 3A. (B) Similarly, the forward primer 20 mer and reverse primer in Fig 3A. (C) Similarly, the forward primer 21 mer and reverse primer in Fig 3A. (D) Similarly, the forward primer 18 mer and reverse primer in Fig 3B. (E) Similarly, the forward primer 17 mer and reverse primer in Fig 3B. (F) Similarly, the forward primer 16 mer and reverse primer in Fig 3B. All photos were taken with a CCD camera attached to BioDoc-It imaging system (UVP, Upland, CA, USA) under UV-B (302 nm) irradiation. Photo A, B and C were printed out without saving the digital data, therefore photos were scanned and edited. All photos were cropped appropriate. Photo A, B and C were flipped horizontally. (PDF) [file pone.0289506.s001.pdf]
