# Supplementary material for: Photoinduced electron transfer detection method for identifying UGT1A1*28 microsatellites
Source: PLoS One. 2023 Aug 3;18(8):e0289506. doi: 10.1371/journal.pone.0289506 (PMC10399816; doi:10.1371/journal.pone.0289506)
Supplement: Attachment — Submitted filename: Response to Reviewers.docx [file pone.0289506.s002.docx]

-----------------------------------------------------------------------------------------------------------------------

Response to the Reviewer #1

-----------------------------------------------------------------------------------------------------------------------

Thank you for your review of our paper. We have answered your points below.

***The Fig. 5 needs attention and occasionally the edits throughout the manuscript. Once these comments are taken cared, the manuscript is acceptable for publication in this journal.***

Answer: We thank for your helpful suggestion. We have checked all figures and corrected any misalignment of objects and text in the figures. We have also changed all figures from doc format to tiff format according to PLOS ONE guideline. We have checked all figures. Other changes to the manuscript are shown **red bold** in the revised manuscript.

Once again, thank you for your review and helpful comments.

-----------------------------------------------------------------------------------------------------------------------

Response to the Reviewer #2

-----------------------------------------------------------------------------------------------------------------------

Thank you for your review of our paper. We have answered each your points below. Changes to the manuscript are shown **blue bold** in the revised manuscript.

***1. The author claims that naked-eye inspection can be performed. What does it mean exactly?***

Answer: Thank you for your comment. When a florescence-labeled probe is hybridized to a target single-strand DNA, the fluorescence intensity of the probe decreases when the target DNA has six TA repeats, while the fluorescence intensity of the probe does not decrease when the target DNA has seven TA repeats. This difference in florescence intensity can be observed with the naked-eye. In other words, we can detect the difference in number of TA repeats (*UGT1A1*28*) as the difference in fluorescence intensity with the naked-eye. We have shown this result in Fig 8E.

***2. Is the detection method proposed by the author specific?***

Answer: Thank you for your comment. We are very sorry. We are not sure what "specific" mean. If you mean "specific = original", then the answer is "yes"; We are the first to apply it to *UGT1A1*28* detection, although PeT is a well-known phenomenon. If you mean "specific = limited", the answer is "yes". The probe used in this study is limited to *UGT1A1*28* detection. It can be applied to other microsatellite detection by changing the probe. Alternatively, if "specific = concrete", the answer is "No". We have not applied this method on human. The concern here is that PCR using genomic DNA collected from the subject will not yield a sufficient amount of target DNA. If "specific = precise", answer is "no". Even though this method can identify homozygotes (TA6/TA6 and TA7/TA7). We need to design a better probe for identification of homozygous and heterozygous.

***3. What are the advantages of this method compared with other existing methods?***

Answer: The advantage of this method is that dose not require special (and expensive) equipment or reagents. While other methods require both a thermal cycler and a fluorescent microplate reader, or a real-time PCR device, this method requires only a standard thermal cycler and UV light. We have added this point to the conclusion section (line 510–511).

***4. The format of the references is incomplete, such as references 11 and 26. It is recommended to check carefully.***

Answer: We have corrected reference 11 and 26 in REFERENCES section (line 557 and 606). We have also checked and corrected other references, according to the PLOS ONE guidelines.

Once again, thank you for your review and helpful comments.
